# Spatiotemporal Patterns of Japanese Encephalitis in China, 2002–2010

**DOI:** 10.1371/journal.pntd.0002285

**Published:** 2013-06-20

**Authors:** Li-Ya Wang, Wen-Yi Zhang, Fan Ding, Wen-Biao Hu, Ricardo J. Soares Magalhaes, Hai-Long Sun, Yi-Xing Li, Wen Zou, Yong Wang, Qi-Yong Liu, Shen-Long Li, Wen-Wu Yin, Liu-Yu Huang, Archie C. A. Clements, Peng Bi, Cheng-Yi Li

**Affiliations:** 1 Institute of Disease Control and Prevention, Academy of Military Medical Science, Beijing, People′s Republic of China; 2 Chinese Center for Disease Control and Prevention, Beijing, People′s Republic of China; 3 School of Population Health, Infectious Disease Epidemiology Unit, The University of Queensland, Brisbane, Australia; 4 Discipline of Public Health, University of Adelaide, Adelaide, Australia; Oswaldo Cruz Foundation, Brazil

## Abstract

**Objective:**

The aim of the study is to examine the spatiotemporal pattern of Japanese Encephalitis (JE) in mainland China during 2002–2010. Specific objectives of the study were to quantify the temporal variation in incidence of JE cases, to determine if clustering of JE cases exists, to detect high risk spatiotemporal clusters of JE cases and to provide evidence-based preventive suggestions to relevant stakeholders.

**Methods:**

Monthly JE cases at the county level in mainland China during 2002–2010 were obtained from the China Information System for Diseases Control and Prevention (CISDCP). For the purpose of the analysis, JE case counts for nine years were aggregated into four temporal periods (2002; 2003–2005; 2006; and 2007–2010). Local Indicators of Spatial Association and spatial scan statistics were performed to detect and evaluate local high risk space-time clusters.

**Results:**

JE incidence showed a decreasing trend from 2002 to 2005 but peaked in 2006, then fluctuated over the study period. Spatial cluster analysis detected high value clusters, mainly located in Southwestern China. Similarly, we identified a primary spatiotemporal cluster of JE in Southwestern China between July and August, with the geographical range of JE transmission increasing over the past years.

**Conclusion:**

JE in China is geographically clustered and its spatial extent dynamically changed during the last nine years in mainland China. This indicates that risk factors for JE infection are likely to be spatially heterogeneous. The results may assist national and local health authorities in the development/refinement of a better preventive strategy and increase the effectiveness of public health interventions against JE transmission.

## Author Summary

Japanese encephalitis (JE) is a mosquito-borne disease, which primarily occurs in rural and suburban areas of Southeast Asia and the Western Pacific region. JE still remains a significant public health problem in mainland China, with approximately 50% of global cases annually. Few studies have explored the spatiotemporal patterns of JE cases in China. Here we reported the results of Local Indicators of Spatial Association and spatial scan statistics of JE cases in mainland China at the county level during the four periods: 2002; 2003–2005; 2006; 2007–2010. The primary spatiotemporal cluster of JE was detected in Southwestern China between July and August, with the geographical range of JE transmission increasing over the past years. The results of LISA and spatial scan statistics were consistent which indicates that these methods are reliable and could have wider applications in the fields of disease surveillance and management in China, particularly in the surveillance and monitoring of other vector-borne diseases. These findings may assist in informing prevention and control strategies and increase the effectiveness of public health interventions against JE transmission.

## Introduction

Japanese encephalitis (JE) is a mosquito-borne disease, which primarily occurs in rural and suburban areas of Southeast Asia and the Western Pacific region [1]–[3]. Japanese encephalitis virus (JEV) is transmitted in an enzootic cycle among mosquitoes and vertebrate amplifying hosts, primarily in domestic pigs and ardeid birds naturally [4]. Vectors, primarily *Culex tritaeniorhynchus*, are abundant in rural areas where their larvae breed in rice paddies and pools of stagnant water. Humans are a dead-end host and get infected when bitten by infected mosquitoes [5]. A recent literature review estimated that annually a total of 68,000 JE cases are reported worldwide [6]. JE cases present a wide spectrum of clinical manifestations, which vary from non-specific febrile flu-like illness to severe clinical manifestations including behavioral abnormality, alteration in sensorium and respiratory paralysis [7].

Historically, large human outbreaks of JE were observed in China in the 1960s (up to 15,000 JE cases were reported) and 1970s (up to 17,000 JE cases were notified) [8]. The number of JE cases has declined substantially from 20.92/100,000 in 1971 to 0.23/100,000 in 2008 since the beginning of a nationwide immunization programme in the early 1970's [9], [10]. However, JE still remains a significant public health problem in mainland China, with approximately 50% of global cases annually [6]. In recent years, evidence suggests that JEV has expanded its geographic limits within China. For example, JEV has been identified in mosquitoes and pigs in Tibet, where was previously believed to be free of JE because of its altitude [11]. Because of the high mortality and protracted severe sequelae, JE still causes a severe health burden [7], [12]–[14].

In the past decades, spatiotemporal analysis techniques have been widely used in infectious disease surveillance and outbreak investigation [15]–[17]. It is used to visualize epidemiological data, detect and evaluate hotspots or clusters and improve surveillance and efficient vector control programmes. Studies using spatiotemporal analysis have been widely used in the field of disease mapping such as Sleeping Sickness [18], dengue fever [19], [20], and malaria [21], [22], however its application in JE has been minimal [23]. Few studies have explored the spatiotemporal patterns of JE cases in China [24], [25].

In the absence of specific treatment for the disease and ineffective and unskilled vector control and management of the amplifying hosts in resource-poor regions [26], interventions need to target vaccination to areas most in need. To inform the efficient targeting of vaccination programmes, it is important to characterize the spatiotemporal pattern of JE cases. Our study was designed to partly address these gaps in knowledge and aimed to describe the nationwide JE epidemic status throughout China, to explore the presence of spatial and seasonal patterns of JE cases, to identify the spatiotemporal clusters of JE cases at the county level and hence to provide evidence-based suggestions for policy-makers and service providers for disease control and prevention.

## Materials and Methods

### Ethics statement

The study was approved by the Ethics Committee of Beijing Institute of Disease Control and Prevention. In this study, all the data analyzed were anonymized for the confidentiality.

### Data collection and management

Data on monthly JE cases from January 2002 to December 2010 were collected through the China Information System for Diseases Control and Prevention (CISDCP). In this study, all JE cases were confirmed according to the unified diagnostic criteria issued by the Ministry of Health of the People's Republic of China [27]. The case definition for JE consists of an individual who lived in an epidemic area during the vector-biting season or travelled to an epidemic area within 25 days prior to illness onset, showing clinical manifestations such as abrupt onset of fever, headache, vomiting, convulsions or drowsiness or movement and consciousness disorders and with one of the following: JEV-specific IgM antibody in a single sample of cerebrospinal fluid (CSF) or serum, JE virus antigens, a 4-fold rise in JE virus-specific antibody, JE virus genome in samples by PCR, or isolated JE virus. Demographic information for each county was collected from the National Bureau of Statistics of China. For the purpose of performing spatial analysis, the county was considered as the spatial unit of analysis and a county-level vector map was acquired from National Administration of Surveying, Mapping and Geoinformation.

### Cluster analysis

Local Indicators of Spatial Association (LISA) was used to describe the spatial pattern of JE incidence clusters on the county level during the study periods. LISA was used to identify significant hot spots (High-High), cold spots (Low-Low), and spatial outliers (High-Low and Low-High) by calculating local Moran's *I* between a given location and the average of neighboring values in the surrounding locations[23], [28], [29]. Significance of clusters was measured by Z score, based on the randomization null hypothesis computation. A high positive Z score indicates that the surroundings have spatial clusters (High-High: high values spatial clusters or Low-Low: low values spatial clusters) and a low negative Z score indicates spatial outliers (High-Low: high values surrounded with low values or Low-High: Low values surrounded with high values) [30]. To identify the spatial patterns of JE, LISA analysis were performed independently for the annual average incidence of JE on the county level in each period using ArcGIS software (version 9.3, ESRI, Redlands, CA).

We also evaluated the dataset for presence of high risk space-time clusters using SaTScan software (version 9.1.1), which implements Kulldorff's spatiotemporal scan statistic [31]. Cases files, population files, and coordinate files (centroids of counties) were generated in comma delimited format for analysis. We fitted a discrete Poisson model and using a maximum temporal cluster size of 10% of the study period in the temporal window and the maximum spatial cluster size of 5% of the population at risk in the spatial window to identify space-time clusters. The variable-sized elliptic window scanned for clusters with high rates noting the number of observed and expected inside the window. The primary cluster and secondary clusters were detected through the log likelihood ratio (LLR) test [32]. Significance of clusters was evaluated using Monte Carlo simulation, generating 999 random simulations to obtain P-values. The null hypothesis of a spatiotemporally random distribution was rejected when the P-value was<0.05.

## Results

### Descriptive analysis

**Figure 1:**
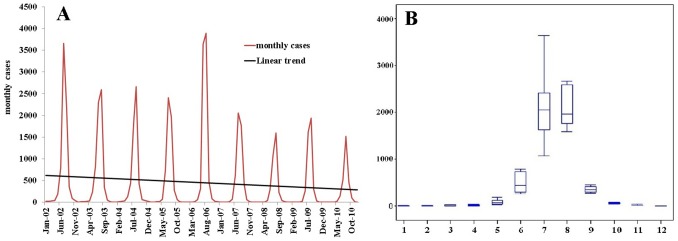
Temporal distribution patterns of JE cases in mainland China. (A) The figure shows epidemic curve of monthly JE cases from 2002–2010; (B) The seasonal epidemic patterns of JE distribution. The bottom and top of the box indicates the lower quartile (P_25_) and the upper quartile (P_75_) respectively; the line in the middle of the box represents the median value; the bottom and top line is minimum and maximum respectively.

A total of 48,892 cases were reported in 1992 counties during 2002–2010 in mainland China in a total of 2,922 counties. Figure 1A describes the monthly distribution of JE cases and its linear trend during 2002–2010. JE cases had a significant seasonal peak with 80.14% cases occurring in July-August (Figure 1B). The annual incidence varied from 0.65/100,000 in 2006 to 0.21/100,000 in 2010. JE showed seasonality with lower peaks: a decreasing trend from 2002 to 2005, peaking in 2006 and then fluctuating then onwards (Figure 1 A). Considering two peaks each 3 or 4 years during the time span of 9 years, we explore the spatiotemporal pattern of the years with large outbreaks (2002 and 2006), the larger peaks from 2003–2005, and the small number of cases (2007 onwards).

**Figure 2:**
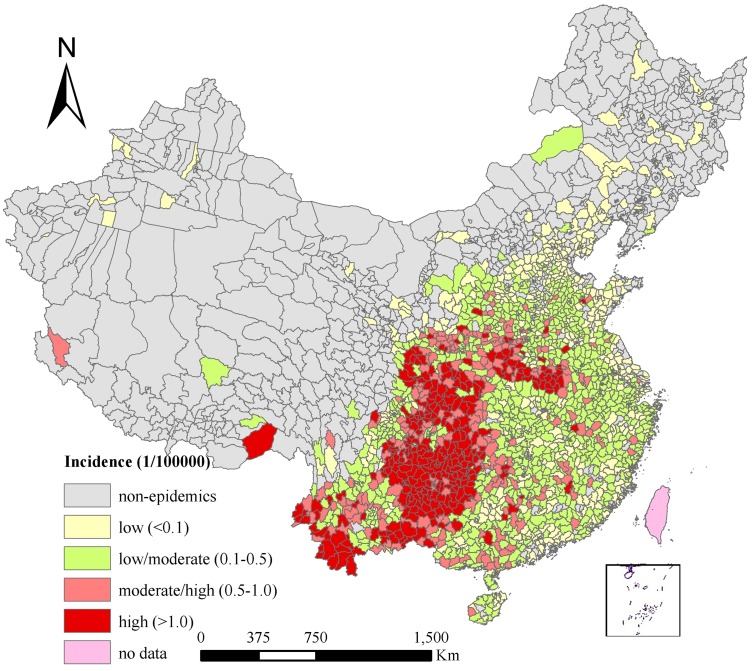
Annual average incidence of JE in mainland China.

Figure 2 describes the spatial distribution of annual average JE incidence at the county level in China over the study period. JE incidence varied among different counties ranging from 0 to 6.41 per 100,000 persons. Group A included non-epidemic areas, with 31.83% of counties covering 62.81% of the total land and 19.16% of the total population; Group B represented low-epidemic areas (with an annual average incidence <0.1/100,000), including 19.30% counties covering 9.04% of the total land and 24.73% of the total population; Group C included low/moderate-epidemic areas (with an annual average incidence ranging from 0.1–0.5/100,000), with 29.77% of counties covering 15.09% of the total land and 34.68% of the total population; Group D included high/moderate-epidemic areas (with an annual average incidence ranging from 0.5–1/100,000), including 9.21% of counties covering 5.78% of the total land and 10.40% of the total population; and Group E being high-epidemic areas (annual average incidence >1/100,000), including 9.89% counties covering 7.29% of the total land and 11.03% of the total population in China. The at-risk population per county varied from 9,649 to 7,048,255 among the counties with JE cases, while in the non-epidemic areas the at-risk population per county varied from 2,123 to 1,241,857.

### Cluster analysis

**Figure 3:**
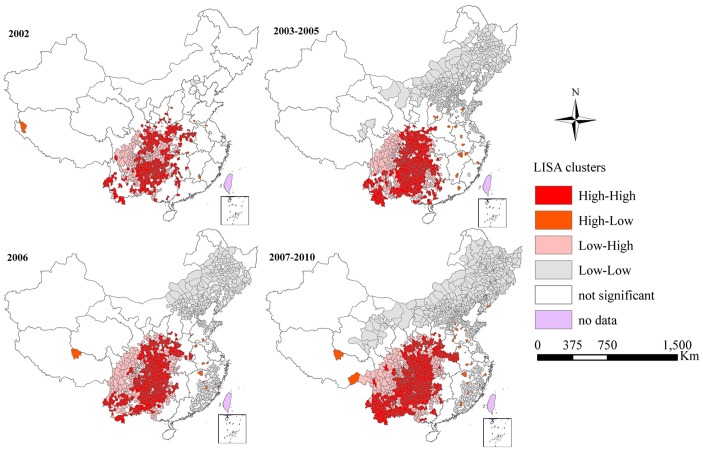
LISA cluster maps for JE incidence in mainland China. LISA spatial cluster map shows the center of the cluster in color. High-High indicates a statistically significant cluster of high JE incidence values; Low-Low indicates a cluster of low JE incidence values; High-Low represents high JE incidence values surrounded with low JE incidence values; Low-High represents low JE incidence values surrounded with high JE incidence values.

LISA analysis of JE epidemics identified foci mainly concentrated in Southwestern China, with an expanding trend to Central China (Figure 3). The shift of hot spots (High-High) and outliers (High-Low) can be observed during the four periods (Table 1). Annual average incidence of High-High cluster had decreased from 3.41/100,000 in 2002 to 1.16/100,000 in 2007–2010. However, the proportion of counties in High-High cluster had increased from 11.09% counties in 2002 to 15.26% counties in 2007–2010. The increasing trend of the proportion of potential infection population was also observed in hotspots clusters, with 12.39% in 2002 to 16.33% in 2007–2010. High-Low outliers were sporadically distributed in Southeastern China in 2002 with 7 counties, increased to 20 counties in 2003–2005, then decreased to 13 counties in 2006, and increased to 21 counties in 2007–2010 (Figure 3).

**Table 1:** JE incidence, proportion of counties, population and cases in spatial clusters, mainland China.

Clusters	Incidence^*^ (1/100000)	%Counties	% Population	% Area
2002				
HH	3.41	11.09	12.39	7.59
HL	3.23	0.24	0.21	0.27
LH	0.004	5.89	5.01	4.44
LL	0	0.07	0.06	0.15
2003–2005				
HH	2.16	12.53	13.84	9.24
HL	1.26	0.68	1.03	0.37
LH	0.05	6.61	4.61	4.83
LL	0.01	16.22	15.61	17.69
2006				
HH	2.98	14.27	14.90	9.49
HL	1.73	0.44	0.58	0.46
LH	0.05	7.43	5.68	6.41
LL	0.002	14.58	14.50	14.70
2007–2010				
HH	1.16	15.26	16.33	10.53
HL	1.04	0.72	0.71	0.57
LH	0.05	5.44	4.03	5.22
LL	0.005	20.23	17.78	23.67

Incidence^*^: annual average incidence, calculated using yearly counts of JE cases as a numerator and population size in the middle of each period as a denominator;

% Counties: calculated using number of counties in each spatial cluster as a numerator and total number of counties as a denominator;

HH: High-High, a statistically significant cluster of high JE incidence values; LL: Low-Low, a cluster of low JE incidence values; HL: High-Low, high JE incidence values surrounded with low JE incidence values; LH: Low-High, low JE incidence values surrounded with high JE incidence values.

**Figure 4:**
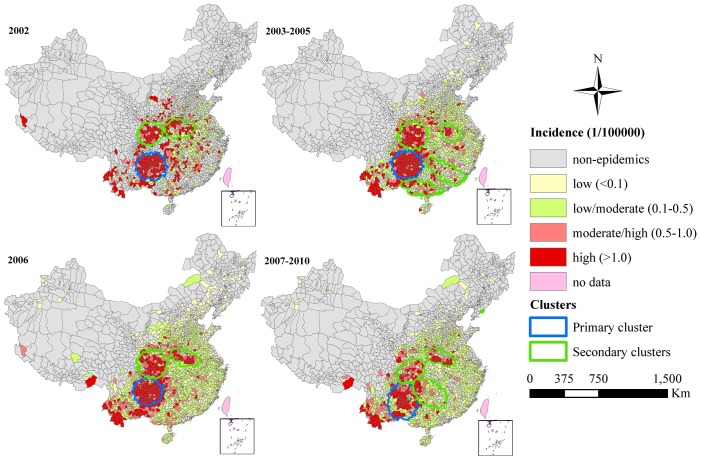
Space-time clusters and annual average incidence of JE cases in mainland China.

Figure 4 shows the distribution of annual average JE incidence and spatiotemporal clusters in China. During the four periods under analysis, the primary cluster of JE occurred in Southwestern China, where the geographic extent was 119, 125, 133, and 144 counties respectively. The relative risk (RR) of JE infection for population inside the primary cluster compared to those outside the cluster ranged from 38.59 in July–August 2007 to 63.17 in July 2002. The highest RR of the primary cluster was identified during July 2002, including 92 counties in Guizhou Province, 9 counties in Sichuan Province, 5 counties in Yunnan Province (Figure 4). In addition, the primary cluster was temporally concentrated during July 2002, 2003–2005, August 2006, and July–August 2007–2010 (Table 2). The identified primary clusters covered 35.05%, 77.59%, 35.81%, and 30.17% of cases and accounted for 3.88%, 4.20%, 4.79%, and 4.97% of the total population in each clustering time frame respectively (Table 3). Secondary clusters of JE cases were mainly located in Southern and Central China. The RR of the secondary clusters varied from 7.97 to 69.02. The highest RR of secondary clusters was identified in Donggang County, Liaoning Province, where 11 cases were unexpectedly notified in September 2007. The 2nd RR of secondary clusters was identified during August 2002, including 46 counties in Sichuan Province, 19 counties in Shaanxi Province, and 7 counties in Gansu Province.

**Table 2:** Space-time clusters of JE defined by using spatial scan statistic, mainland China*.

Clusters	E_Minor	E_Major	E_Angle	E_Shape	Time frame	No. Counties	No. Obs	No. Exp	LLR	RR
2002										
1<$>\scale 60%\raster="rg1"<$>	233	233	n/a	1.0	2002/7	119	1281	24.44	3928.80	63.17
2#	202	202	n/a	1.0	2002/8	74	712	19.06	1918.26	41.21
3	166	249	n/a	1.5	2002/7	98	568	31.07	1133.57	19.71
2003–2005										
1	240	240	n/a	1.0	2003/7	125	1787	42.02	5045.62	47.19
2	244	244	n/a	1.0	2005/7–2005/8	105	1053	44.81	2345.45	24.92
3	127	190	−45°	1.5	2005/7	66	399	19.01	838.73	21.45
4	230	460	−30°	2.0	2003/5–2003/6	84	380	44.43	511.59	9.02
5	131	393	−40°	3.0	2003/6–2003/7	119	383	49.00	456.73	7.97
2006										
1	228	228	n/a	1.0	2006/8	133	1393	34.59	3905.00	47.97
2	243	243	n/a	1.0	2006/8	114	923	35.15	2176.81	29.34
3	126	252	−30°	2.0	2006/7	88	585	35.84	1102.73	17.46
2007–2010										
1	220	330	90°	1.5	2007/7–2007/8	144	1153	32.24	3045.52	38.59
2	126	252	−30°	2.0	2009/7–2009/8	98	735	32.46	1607.06	23.74
3	170	255	45°	1.5	2008/7–2008/8	91	688	31.86	1471.96	22.56
4	197	295	−45°	1.5	2007/7–2007/8	146	340	32.42	494.64	10.70
5	0	0	n/a	1.0	2007/9	1	11	0.16	35.73	69.02

* Significant clusters with P<0.01;

<$>\scale 60%\raster="rg1"<$>1: Primary cluster;

# 2–5: Secondary clusters; E_Minor: Semiminor axis of ellipse; E_Major: Semimajor axis of ellipse; E_Angle: the angle between the horizontal line and the semimajor axis of the ellipse; E_Shape: E_Major: E_Minor; No. Counties: number of counties within clusters; No. Obs: number of observed cases; No. Exp: number of expected cases; LLR: log likelihood ratio; RR: relative risk for the cluster compared with the rest of the country.

**Table 3:** JE incidence, proportion of population and cases in spatiotemporal clusters, mainland China.

Clusters	Time frame	Incidence^*^ (1/100000)	% Population	% Case
2002				
1<$>\scale 60%\raster="rg1"<$>	2002/7	2.54	3.88	35.05
2#	2002/8	1.81	3.03	32.87
3	2002/7	0.88	4.94	15.54
2003–2005				
1	2003/7	3.27	4.20	77.59
2	2005/7–2005/8	1.81	4.48	24.06
3	2005/7	0.81	3.80	16.56
4	2003/5–2003/6	0.78	3.73	35.41
5	2003/6–2003/7	0.59	4.98	12.19
2006				
1	2006/8	2.23	4.79	35.81
2	2006/8	1.46	4.87	23.73
3	2006/7	0.91	4.97	16.07
2007–2010				
1	2007/7–2007/8	1.79	4.97	30.17
2	2009/7–2009/8	1.13	5.00	20.64
3	2008/7–2008/8	1.08	4.91	25.73
4	2007/7–2007/8	0.52	4.99	8.90
5	2007/9	1.67	0.05	2.68

<$>\scale 60%\raster="rg1"<$>1: Primary cluster;

# 2–5: Secondary clusters;

Incidence^*^: JE incidence during the clustering time.

## Discussion

The results of this study indicate that there was a significant spatiotemporal heterogeneity of JE throughout mainland China during 2002–2010, in that JE reported cases were clustered during the four periods under analysis. Both LISA and spatial scan statistics analysis identified similar clusters, mainly concentrated in Southwestern China and the geographical range of JE transmission increased over the study period. This concentration maybe associated with the distribution of rice-planting areas, the extent of pig rearing and the proportion of the rural population [13], [33], [34]. Rice-planting areas are man-made vectors breeding sites that are preferred by JE vectors [35]. The proportion of the rural population is usually associated with levels of poverty and the availability of funds for JE control and prevention [36]. Non-epidemic areas were mainly distributed in the North and Western regions, where the disease was probably not autochthonous, although researchers have isolated JEV from mosquito samples and detected JEV antibody in local population and pigs [37].

Spatial scan statistics, considering both spatial and temporal features, are commonly used in disease surveillance for geographical cluster detection and evaluation, for which they have been shown to have good statistical power [38]. This allows health officials to investigate disease outbreaks whether due to environmental factors, differences in behavioural factors, the transmission of an infectious agent, and the genetic makeup of the population or not [39]. The methods used in the study were previously used in Nepal in a study which showed that the distribution of JE cases had shifted, with clusters found in the central hill areas [23]. Local indicators of spatial association identify patterns in geographic units which deviate from the average of neighboring values in the surrounding locations [40]. LISA, a spatial cluster analysis, has a better chance of detecting true cluster areas with low false-positive rates especially performing well on outlier detection [41]. Elliptic version of the spatial scan statistic rather than commonly used circular version was selected in our study because it has proven to be more appropriate at detecting whether regular shapes or irregular shapes for most of situations [31], [40]–[42]. Our study identified circular clusters (e.g. primary clusters in 2002, 2003–2005, and 2006) and irregular shapes, indicating elliptic scan window should be preferentially used when the shape of the cluster is not known. The results of LISA and spatial scan statistics were consistent which indicates that these methods are reliable and could have wider applications in the fields of disease surveillance and management in China, in particular to the surveillance and monitoring of other vector-borne diseases [19]–[22].

Overall, our results show that JE cases were widespread but relatively concentrated in rural areas in China, especially in Southwestern China. Our findings indicate that preventative strategies for JE, including boosting existing surveillance, financial support from central and provincial governments, infrastructure redevelopment and in-house workforce training should be particularly focused to counties in Southwestern China identified in this study.

In addition to contributing to the scientific advancement of JE epidemiology in China, the results from this study also provide important evidence to health authorities, policy-makers and public health practitioners and other service providers to improve JE control. For example, prevention and control measures including service guideline establishment and refinement, disease and vector/host surveillance, immunization program implementation, local vector control, health education and promotion campaigns, community engagement and environmental management, should focus on the high risk areas identified in our study. Targeting of prevention strategies at high-risk clusters is likely to increase the program's effectiveness. Individuals in highest risk areas should be informed of the risk and the possibilities for risk reduction.

The results of this study have to be interpreted in light of the studies' limitations: firstly, the data are from a passive surveillance system, which means that some cases of JE might go underreported because of their sub-clinical symptoms [12], [13], [43]. Secondly, we used an elliptic scan window in the spatial scan statistics. Although the elliptic scan algorithm searches more zones than a circular scan window, it requires more computational time. In addition, the elliptic scan window has been reported to perform poorly compared to the circular window at detecting for large clusters, as it may select long and narrow string of noncontiguous areas [39].

## 

### Conclusion

This study described the spatiotemporal patterns of JE in mainland China and identified spatial and temporal high risk clusters at the county level over the last 9 years, with important public health implications for targeting JE control in the country. Further studies are needed to explore the role of the physical environment (e.g. landscape and climate) and social environment (e.g. human movement, farming activities, housing conditions and personal health behavior environment), population immunity, mosquito control measures in driving the spatiotemporal distribution identified in this study.

## References

[1] Elias C, Okwo-Bele JM, Fischer M (2009) A strategic plan for Japanese encephalitis control by 2015. Lancet Infect Dis 9: 7. 19095192 10.1016/S1473-3099(08)70290-1

[2] Senior K (2008) Is Japanese encephalitis control achievable by 2013? Lancet Infect Dis 8 9: 534.

[3] van den Hurk AF, Ritchie SA, Mackenzie JS (2009) Ecology and geographical expansion of Japanese encephalitis virus. Annu Rev Entomol 54: 17–35. 19067628 10.1146/annurev.ento.54.110807.090510

[4] Tom S (2006) Control of Janpanese Encephalitis—within our grasp? N ENGL J MED 355: 869–871. 16943399 10.1056/NEJMp058263

[5] Misra UK, Kalita J (2010) Overview: Japanese encephalitis. Prog Neurobiol 91: 108–120. 20132860 10.1016/j.pneurobio.2010.01.008

[6] Campbell GL, Hills SL, Fischer M, Jacobson JA, Hoke CH, et al (2011) Estimated global incidence of Japanese encephalitis: a systematic review. Bull World Health Organ 89: 766–774E, 766-774, 774A-774E. 22084515 10.2471/BLT.10.085233 PMC3209971

[7] Ooi MH, Lewthwaite P, Lai BF, Mohan A, Clear D, et al (2008) The epidemiology, clinical features, and long-term prognosis of Japanese encephalitis in central sarawak, malaysia, 1997–2005. Clin Infect Dis 47: 458–468. 18616397 10.1086/590008

[8] Zheng YY, Li MH, Wang HY, Liang GD (2012) Japanese encephalitis and Japanese encephalitis virus in mainland China. Rev Med Virol 22: 301–322. 22407526 10.1002/rmv.1710

[9] Gao XY, Nasci R, Liang G (2010) The neglected arboviral infections in mainland China. PLoS Negl Trop Dis 4: e624. 20436960 10.1371/journal.pntd.0000624 PMC2860493

[10] Ministry of Health (2009) The Ministry of Health announced the 2008 national notifiable infectious diseases. Available: http://www.moh.gov.cn/publicfiles/business/htmlfiles/mohbgt/s3582/200902/39079.htm.

[11] Li YX, Li MH, Fu SH, Chen WX, Liu QY, et al (2011) Japanese encephalitis, Tibet, China. Emerg Infect Dis 17: 934–936. 21529419 10.3201/eid1705.101417 PMC3321773

[12] Yin Z, Wang H, Yang J, Luo H, Li Y, et al (2010) Japanese encephalitis disease burden and clinical features of Japanese encephalitis in four cities in the People's Republic of China. Am J Trop Med Hyg 83: 766–773. 20889863 10.4269/ajtmh.2010.09-0748 PMC2946740

[13] Erlanger TE, Weiss S, Keiser J, Utzinger J, Wiedenmayer K (2009) Past, present, and future of Japanese encephalitis. Emerg Infect Dis 15: 1–7. 19116041 10.3201/eid1501.080311 PMC2660690

[14] Wang L, Zhang W, Ding F, Sun H, Yu S, et al (2013) Deaths associated with Japanese encephalitis, china, 2005–2010. Clin Infect Dis 56: 752. 23243182 10.1093/cid/cis1012

[15] Fosgate G, Carpenter T, Chomel B, Case J, DeBess E, et al (2002) Time-space clustering of human Brucellosis, California, 1973–1992. Emerg Infect Dis 8: 672–678. 12095433 10.3201/eid0807.010351 PMC2730319

[16] Garcia H, Gonzalez A, Evans C, Gilman R (2003) Taenia solium cysticercosis. Lancet Infect Dis 362: 547–556. 10.1016/S0140-6736(03)14117-7 PMC3103219 12932389

[17] Naish S, Hu W, Mengersen K, Tong S (2011) Spatio-temporal patterns of Barmah Forest virus disease in Queensland, Australia. PloS One 6 10: e25688 doi:10.1371/journal.pone.0025688 22022430 10.1371/journal.pone.0025688 PMC3192738

[18] Berrang-Ford L, Berke O, Abdelrahman L, Waltner-Toews D, McDermott J (2006) Spatial analysis of Sleeping Sickness, Southeastern Uganda, 1970–2003. Emerg Infect Dis 12: 813–820. 16704843 10.3201/eid1205.051284 PMC3293436

[19] Banu S, Hu W, Hurst C, Guo Y, Islam MZ, et al (2012) Space-time clusters of dengue fever in Bangladesh. Trop Med Int Health 17: 1086–1091. 22809407 10.1111/j.1365-3156.2012.03038.x

[20] Li Z, Yin W, Clements A, Williams G, Lai S, et al (2012) Spatiotemporal analysis of indigenous and imported dengue fever cases in Guangdong province, China. BMC Infect Dis 12: 132. 22691405 10.1186/1471-2334-12-132 PMC3412724

[21] Clements AC, Barnett AG, Cheng ZW, Snow RW, Zhou HN (2009) Space-time variation of malaria incidence in Yunnan province, China. Malar J 8: 180. 19646240 10.1186/1475-2875-8-180 PMC2724544

[22] Zhang W, Wang L, Fang L, Ma J, Xu Y, et al (2008) Spatial analysis of malaria in Anhui province, China. Malar J 7: 206. 18847489 10.1186/1475-2875-7-206 PMC2572066

[23] Impoinvil DE, Solomon T, Schluter WW, Rayamajhi A, Bichha RP, et al (2011) The spatial heterogeneity between Japanese encephalitis incidence distribution and environmental variables in Nepal. PLoS One 6: e22192. 21811573 10.1371/journal.pone.0022192 PMC3141013

[24] Bi P, Zhang Y, Parton KA (2007) Weather variables and Japanese encephalitis in the metropolitan area of Jinan city, China. J Infect 55: 551–556. 17714787 10.1016/j.jinf.2007.07.004

[25] Wang H, Li Y, Liang X, Liang G (2009) Japanese encephalitis in mainland china. Jpn J Infect Dis 62: 331–336. 19762980

[26] Marfin AA, Gubler DJ (2005) Japanese encephalitis: the need for a more effective vaccine. Lancet 366: 1335–1337. 16226596 10.1016/S0140-6736(05)67543-5

[27] Ministry of Health (2006) National surveillance plan for Japanese encephalitis. Available: http://www.moh.gov.cn/open/uploadfile/2006627153210951.doc.

[28] Hamer GL, Chaves LF, Anderson TK, Kitron UD, Brawn JD, et al (2011) Fine-scale variation in vector host use and force of infection drive localized patterns of West Nile virus transmission. PLoS One 6: e23767. 21886821 10.1371/journal.pone.0023767 PMC3158794

[29] Kamdem C, Fouet C, Etouna J, Etoa FX, Simard F, et al (2012) Spatially explicit analyses of anopheline mosquitoes indoor resting density: implications for malaria control. PLoS One 7: e31843. 22348131 10.1371/journal.pone.0031843 PMC3279417

[30] Anselin L (1995) Local indicators of spatial association - LISA. Geographical Analysis 27: 93–115.

[31] Kulldorff M (1997) A spatial scan statistic. Commun Stat Theory Methods 26: 1481–1496.

[32] Kulldorff M (2010) SaTScan User Guide for version 9.0. Available: http://wwwsatscanorg.

[33] Cao M, Feng Z, Zhang J, Ma J, Li X (2010) Contextual risk factors for regional distribution of Japanese encephalitis in the People's Republic of China. Trop Med Int Health 15: 918–923. 20561307 10.1111/j.1365-3156.2010.02563.x

[34] Keiser J, Maltese MF, Erlanger TE, Bos R, Tanner M, et al (2005) Effect of irrigated rice agriculture on Japanese encephalitis, including challenges and opportunities for integrated vector management. Acta Trop 95: 40–57. 15878762 10.1016/j.actatropica.2005.04.012

[35] Diagana M, Preux PM, Dumas M (2007) Japanese encephalitis revisited. J Neurol Sci 262: 165–170. 17643451 10.1016/j.jns.2007.06.041

[36] Zhu CG, Su SS, Li ZJ (2008) The influencing factors and spatial distribution of population urbanization in China. Geographical Research 27: 13–23.

[37] Li MH, Fu SH, Chen WX, Wang HY, Guo YH, et al (2011) Genotype V Japanese Encephalitis virus is emerging. PLoS NTD 5: e1231. 10.1371/journal.pntd.0001231 PMC3130007 21750744

[38] Kulldorff M, Tango T, Park P (2003) Power comparisons for disease clustering tests. Comput Stat Data An 42: 665–684.

[39] Kulldorff M, Huang L, Pickle L, Duczmal L (2006) An ellipit spatial scan statistic. Statist Med 25: 3929–43. 10.1002/sim.2490 16435334

[40] Huang L, Pickle LW, Das B (2008) Evaluating spatial methods for investigating global clustering and cluster detection of cancer cases. Stat Med 25: 5111–5142. 10.1002/sim.3342 PMC2575694 18712778

[41] Jackson MC, Huang L, Luo J, Hachey M, Feuer E (2009) Comparision of tests for spatial heterogeneity on data with global clustering patterns and outliers. Int J Health Geogr 8: 55. 19822013 10.1186/1476-072X-8-55 PMC2770045

[42] Duczmal L, Kullodrff M, Lan H (2006) Evaluation of spatial scan statistics for irregular shaped clusters. Journal of Computional & Graphical Statistics 2: 15.

[43] WHO (2009) Fourth Biregional Meeting on the control of Japanese Encephalitis. Available: http://www.searo.who.int/catalogue/2005-2011/pdf/Japanese%20encephalitis/sea-immun-59.pdf.

